# Trends in EU regulatory assessment of oncology medicines

**DOI:** 10.1016/j.esmoop.2026.107732

**Published:** 2026-05-21

**Authors:** T. Pabsch, F. Pignatti, F. Day, B. Sepodes, H. Enzmann

**Affiliations:** 1 Executive Department European Union and International Affairs, Federal Institute for Drugs and Medical Devices (BfArM), Bonn, Germany; 2 Oncology & Haematology Office, European Medicines Agency, Amsterdam, The Netherlands; 3 Therapeutic Areas Department, European Medicines Agency, Amsterdam, The Netherlands; 4 Faculdade de Farmácia, Universidade de Lisboa, Lisbon, Portugal; 5 Laboratory of Systems Integration Pharmacology, Clinical and Regulatory Science, Research Institute for Medicines of the University of Lisbon (iMED.ULisboa), Lisbon, Portugal

**Keywords:** European Medicines Agency, centralised procedure, oncology medicines, regulatory assessment, regulatory complexity

## Abstract

**Background:**

During marketing authorisation applications (MAAs), regulators assess extensive data, identifying issues and uncertainties requiring clarification before a medicine reaches the market. Despite structured reviews, quantitative evaluation of regulatory assessment complexity remains limited, constraining systematic insights into challenges of assessments.

**Materials and methods:**

Focusing on oncology, this study analysed the regulatory complexity of MAAs submitted to the European Medicines Agency, based mainly on the number of distinct issues raised during initial dossier assessment. Issues were disaggregated into quality, nonclinical, and clinical domains to identify primary drivers of complexity, temporal trends, and product- or pathway-specific differences. As a complementary measure, European public assessment report (EPAR) length was examined as the final public documentation of the assessment outcome.

**Results:**

Between 2015 and 2024, the number of issues increased substantially across all domains (+60%), most prominently clinical issues, driven largely by efficacy-related concerns. Advanced therapy medicinal products generated more quality-related issues, reflecting complex manufacturing, whereas tumour-agnostic indications elicited more clinical issues, consistent with paradigm-shifting trial designs. Conditional marketing authorisation, introduced to manage uncertainty, was associated with higher overall issue counts. Trends in EPAR length paralleled those observed for clinical issue counts, indicating that regulatory assessment complexity during initial review is reflected in the final public documentation.

**Conclusions:**

The growing number of distinct issues raised during initial dossier assessment highlights a marked increase in regulatory complexity in oncology MAAs over the past decade. The measures capture both product-specific and systemic procedural influences, providing a quantitative foundation to inform resource allocation, process optimisation, and regulatory strategy.

## Highlights

• EU oncology MAAs (2015-2024) showed a sustained rise in assessment complexity, reflected by an increasing number of issues.
• Issues increased across all domains (+60%), most prominently in the clinical domain.
• EPAR length paralleled clinical issue trends, reflecting increasing assessment complexity.
• The applied metrics capture both product-specific and procedural drivers of regulatory complexity.

## Introduction

With anticancer agents accounting for about one-third of new chemical entity (NCE) approvals in Europe, oncology represents a field of rapid innovation, marked by high scientific complexity and diverse evidence generation in marketing authorisation applications (MAAs).^1^ Advances in targeted therapies, immune-oncology, and biomarker-guided medicines are reshaping therapeutic paradigms,^2^ requiring regulatory assessors to navigate large datasets to safeguard scientific rigour, evaluate benefit–risk profiles, and ensure timely patient access.^3^ Given the high volume of applications, the relative uniformity of clinical endpoints across cancer types, and the emergence of innovative modalities such as antibody–drug conjugates, immune checkpoint inhibitors, tumour-agnostic therapies, and cell-based treatments,^4^, ^5^, ^6^, ^7^ oncology provides a particularly informative setting for studying trends in regulatory assessment, the interaction between evidence complexity and decision making, and the evolution of assessment practices.

In the European Union (EU), the approval of innovative anticancer medicines follows a centralised procedure designed to allow applicant companies to submit a single application for review, and if the legal quality, safety, and efficacy requirements are met, market the medicine and make it available to patients and healthcare professionals throughout the EU.^8^ Applicants submit comprehensive dossiers covering manufacturing, preclinical and clinical data, which are evaluated by scientific committees, relying on the scientific evaluation and resources available to national marketing authorisation bodies, and supported and coordinated by the secretariat of the European Medicines Agency (EMA). Within this framework, the evaluation is coordinated by one committee member designated as rapporteur and, optionally, another as co-rapporteur, who draw up the draft assessment reports, while the remaining committee members contribute through iterative commenting and discussion. The Committee for Medicinal Products for Human Use (CHMP), which is ultimately responsible for the scientific evaluation, adopts an assessment report and issues a scientific opinion that is the basis for the European Commission’s decision on the granting or refusal of the marketing authorisation. The 80-day initial scientific assessment by the rapporteur(s) represents the first important stage of this process, during which the rapporteurs perform the initial detailed assessment of the dossier. This phase results in an assessment report and a list of questions adopted by the committee that consolidates the issues and uncertainties requiring clarification before a marketing authorisation can be granted. The ensuing structured dialogue between regulators and applicants is central to addressing gaps in the evidence base and ensuring that the benefit–risk profile is reliably established.

Despite the structured nature of MAAs, robust indicators capturing the inherent complexity and diverse thematic scope of regulatory evaluations remain limited, constraining systematic analysis. One promising approach lies in the issues raised during the initial assessment. Beyond their procedural function, these issues may present a useful window into the complexities inherent in the assessment of a given application. As the first comprehensive evaluation of the full dossier, they could serve as a proxy for procedural complexity, with their number reflecting a combination of scientific novelty, gaps or ambiguities in the submitted data, as well as the influence of an evolving regulatory framework. Although undoubtedly subject to substantial variability from multiple, difficult-to-disaggregate factors, this overarching metric can provide valuable insights into trends and regulatory challenges across submissions.

This work examines selected procedural metrics, specifically the number of distinct issues raised during initial dossier assessment and the length of the European public assessment report (EPAR), to identify factors influencing regulatory assessment and recurring patterns. Disaggregated by the major scientific domains of quality, nonclinical, and clinical assessment, and their respective subdomains [drug substance and drug product for quality; pharmacokinetics and pharmacodynamics (PK/PD) and toxicology for nonclinical; PK/PD, efficacy, and safety for clinical], they offer the prospect of providing granular insights into assessment complexity and regulatory engagement with innovation and uncertainty.

These metrics may allow characterisation of temporal trends, product- and pathway-related differences, and systemic influences. Such insights can support the interpretation of regulatory decisions, strategic planning, and professional advancement within regulatory agencies.

## Materials and methods

### Overview and data acquisition

This retrospective study covered the period from 2015 to 2024, encompassing a decade of regulatory data. The analysis included oncology medicines for human use, identified based on their anatomical therapeutic chemical classification, that received a positive opinion from the CHMP within the specified timeframe and were granted an initial marketing authorisation in the EU, irrespective of their current regulatory status. Only products containing at least one NCE and authorised through a full application under Article 8(3) of Directive 2001/83/EC, as well as those granted a Conditional Marketing Authorisation (CMA) or a Marketing Authorisation under Exceptional Circumstances (MAUEC), were eligible for inclusion. Accordingly, any generic, biosimilar, and hybrid applications were excluded. Radiopharmaceutical precursors were also excluded due to the absence of a comprehensive clinical dossier. A total of 128 medicines were identified that met the prespecified criteria. The date of the medicine’s positive CHMP opinion was used as the reference time point for all temporal analyses.

Issues raised in the rapporteur’s initial day 80 list of questions (or day 60 in case of accelerated assessment procedures) were extracted. Given procedural changes during the study period, most notably the substitution of the co-rapporteur’s independent assessment report at day 80 with the co-rapporteur’s critique in 2021, analyses focused on issues raised by the rapporteur, as this represents the most stable and comparable metric across time. After extraction and where appropriate, issues were aggregated or disaggregated into their underlying thematic components. This approach was employed to account for heterogeneity in the formulation of compound questions across assessment teams and to allow a more accurate quantification of distinct issues raised. Issues were categorised as relating to quality (Q), nonclinical (NC), or clinical (C) aspects. Other domains, such as those primarily administrative in nature or oriented towards post-authorisation activities (e.g. pharmacovigilance), were excluded from the analysis due to their limited relevance to the initial scientific assessment of benefit and risk. Quality-related issues were further subclassified as referring to either drug substance (DS) or drug product (DP); nonclinical issues were assigned to either nonclinical pharmacokinetics/pharmacodynamics (NP) or toxicology (TX); and clinical issues were further categorised as related to *clinical PK/PD* (CP), efficacy (E), or safety (S). EPARs were accessed via the EMA website, and the number of pages per report was documented. Procedure duration was defined as the time (in days) between the procedural start date and the issuance of the CHMP opinion, based on data from the EMA database.

### Disaggregation factors

Data were disaggregated according to regulatory characteristics to enable comparative analyses:

• Type of marketing authorisation procedure: Applications were classified as either standard marketing applications [Article 8(3) of Directive 2001/83/EC], CMA [Article 14-a of Regulation (EC) No 726/2004], or MAUEC [Article 14(8) of Regulation (EC) No 726/2004].
• Participation in expedited or supportive regulatory pathways: Procedures were further categorised based on participation in accelerated assessment [Article 14(9) of Regulation (EC) No 726/2004] or the EMA Priority Medicines scheme, which provides enhanced regulatory interaction and support.
• Size of the applicant company: Procedures were classified according to the size of the applicant company, defined as inclusion among the 25 largest biomedical companies worldwide by total revenue, based on annual reports from 2024 (or, where unavailable, from 2023), or not (see Supplementary Table S1, available at https://doi.org/10.1016/j.esmoop.2026.107732). This threshold was chosen as a pragmatic cutoff to explore potential associations between company size, as a proxy for regulatory experience and available resources, and characteristics of the marketing authorisation procedure.

Data were further disaggregated by active substance type and indication-related regulatory attributes to evaluate differences on the analysed measures:

• Active pharmaceutical ingredient (API) type: Medicines were classified according to the nature of their API. A distinction was made between chemically synthesised small-molecule products and biologically derived products produced using living systems.
• Advanced therapy medicinal products (ATMPs): Biological products were further subclassified based on their classification by the EMA as ATMPs. ATMPs include gene therapy medicines, somatic cell therapy medicines, and tissue-engineered medicines, as defined under Regulation (EC) No 1394/2007.
• First in class (FIC): Procedures were categorised based on whether the targeted product was considered FIC. For this analysis, a product was defined as FIC if it was the first centrally authorised product in the EU containing an active substance that targets a novel molecular mechanism of action not previously addressed by any other authorised medicine. Products sharing a similar mechanism of action or targeting the same molecular structure as a previously authorised substance were categorised as non-FIC.
• Targeted therapy: Products were classified as targeted therapies (also called personalised medicines) if, at the time of initial MAA, at least one of the applied for indications required the identification of a specific molecular marker. Only products with an explicitly defined biomarker-dependent population in the applied for indication were included in this category. Products approved for broader, biomarker-independent populations were classified as nontargeted therapies.
• Tumour-agnostic indications: Medicines were classified according to whether they had applied for a tumour-agnostic indication at the time of initial marketing authorisation. Tumour-agnostic indications refer to targeted therapies approved based on a specific molecular alteration, irrespective of the tumour’s anatomical origin. Although only a limited number of such medicines were approved, this classification was retained to capture potentially relevant patterns associated with an emerging therapeutic concept.
• Number of indications: The applied indication is counted as one therapeutic indication if it refers to the same disease entity, even if it allows variations in line of therapy, treatment regimen, or biomarker status, provided the target population and therapeutic intent remain broadly consistent. Multiple therapeutic indications (≥2) are counted when the applied indication includes distinct disease entities.
• Orphan designation: Procedures were classified based on whether the medicine had received an orphan designation by the EMA [in accordance with Regulation (EC) No 141/2000].

### Statistical analysis

Results are reported as mean ± standard deviation (SD) to provide an approximate measure of annual complexity, acknowledging that the data were not normally distributed. This approach was deemed appropriate given the objective of describing aggregated trends without underestimating the contribution of outliers, which are relevant for understanding procedural variability and resource implications. In addition, all data are also presented as medians with interquartile ranges in the respective tables or as Supplementary Tables (available at https://doi.org/10.1016/j.esmoop.2026.107732) for all figures. For all statistical comparisons and correlation analyses, nonparametric methods were employed, including Mann–Whitney and Kruskal–Wallis tests for group comparisons. To avoid assumptions inherent to regression models, no curve fitting was applied. Instead, trends were illustrated descriptively based on raw data, and only mean values at the beginning and end of the observation period were compared to prevent overinterpretation through extrapolated model estimates. All analyses were conducted using GraphPad Prism (GraphPad Software Inc., San Diego, California).

## Results

**Figure 1:**
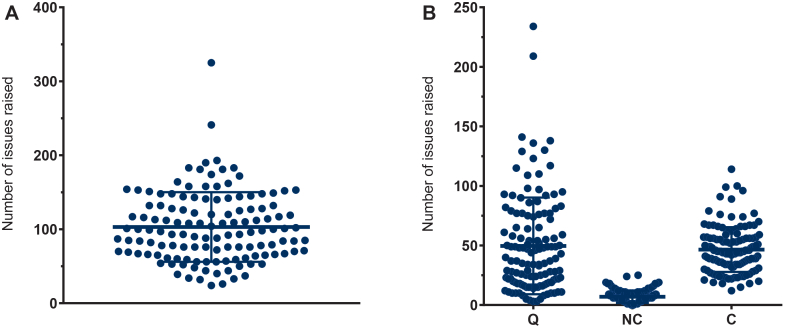
**(A) Number of distinct issues raised per procedure in the rapporteur’s initial assessment report.** 103 ± 47.2; 97.5 (67-132). (B) Distribution of issues by regulatory domain: quality (Q): 49.6 ± 40.6; 38 (19-74.8), nonclinical (NC): 6.9 ± 5.1; 6 (3-10), and clinical (C): 46.6 ± 18.8; 44.5 (34-56). Each dot represents one procedure. Values are presented as mean ± standard deviation; median (interquartile range).

Over a 10-year period (2015-2024), the EU witnessed a range of 7 to 16 positive CHMP opinions annually for NCE oncology marketing authorisation procedures, averaging 12.8 per year (Supplementary Figure S1, available at https://doi.org/10.1016/j.esmoop.2026.107732). Even though the latter half of the observation period showed a modest increase in annual procedures, no clear trend was evident, and the number of procedures remained fairly constant. During the initial assessment round of these procedures, rapporteurs raised a mean of 103.0 distinct issues requiring clarification by applicants (Figure 1A). Notably, the number of issues raised varied significantly, with a minimum of 24 and a maximum of 325, resulting in an SD of 47.2. Upon disaggregation into scientific regulatory domains (Figure 1B), it became apparent that the number of issues raised was roughly evenly distributed between quality (49.6 ± 40.6) and clinical (46.6 ± 18.8) aspects. By contrast, nonclinical issues were raised significantly less frequently, with a mean of 6.9 ± 5.1. A comparison of clinical and quality-related issues suggests a notable difference in variance. Quality-related issues exhibited substantially higher variability, with a small number of procedures receiving an exceptionally high number of issues, while a large proportion of procedures received considerably fewer questions.

The analysis of EPARs for oncology medicines from 2015 to 2024 revealed an average length of 153.3 ± 33.2 pages, as illustrated in Supplementary Figure S2A, available at https://doi.org/10.1016/j.esmoop.2026.107732. The distribution of EPAR lengths exhibited a pattern similar to that of the number of issues raised. However, the overall distribution was more centralised, reflecting a comparatively narrower range and reduced dispersion.

### Procedural factors influencing marketing authorisation assessments

The impact of the application characteristics on procedural metrics was assessed by disaggregating the number of issues raised (overall and by regulatory domain: quality, nonclinical, and clinical) and EPAR length as detailed in Table 1.

**Table 1:** Summary of the number of issues raised (overall and per scientific domain) and EPAR length, disaggregated by regulatory characteristics

	*N*	Number of issues raised	*P*	Thereof quality	*P*	Thereof nonclinical	*P*	Thereof clinical	*P*	Length of EPAR (pages)	*P*
Type of MA			<0.001		0.002		0.906		0.075		0.684
Standard	96	95.2 ± 46.4; 84.5 (64.3-118.5)		43.7 ± 39.02; 29 (16.3-57.5)		6.9 ± 5.3; 6 (3-10)		44.4 ± 17.4; 43 (34-53.8)		152.6 ± 32.6; 144 (130-169)	
CMA	29	125.8 ± 43.1; 126 (101-152)		66.0 ± 41.6; 56 (39-86)		6.8 ± 4.9; 6 (3.5-8)		53.0 ± 22.5; 53 (38-66)		156.3 ± 33.0; 147 (130-185.5)	
MAUEC	3	134.7 ± 22.7; 143 (109-152)		77.3 ± 32.4; 95 (40-97)		5.0 ± 1.7; 6 (3-6)		52.3 ± 12.3; 49 (42-66)		141.0 ± 60.9; 129 (87-207)	
Expedited programmes and support			0.011		0.002		0.037		0.736		0.156
Standard	107	100.1 ± 40.9; 93 (66-132)		46.4 ± 34.4; 37 (19-72)		7.1 ± 4.9; 6 (4-10)		46.6 ± 17.2; 45 (35-56)		154.1 ± 31.9; 147 (131-176)	
Accelerated	8	72.5 ± 36.6; 72.5 (34.3-103.5)		24.5 ± 17.3; 15.5 (11-44.5)		3.7 ± 5.9; 1 (1-4.5)		44.3 ± 29.1; 36.5 (17.3-71.3)		133.8 ± 22.3; 126.5 (116-157)	
PRIME	13	145.8 ± 72.6; 120 (99-182)		91.3 ± 66.8; 77 (43.5-118)		7.0 ± 6.26; 6 (3-8)		47.5 ± 24.8; 42 (28.5-60)		157.3 ± 45.5; 146 (127.5-180.5)	
Applicant size			0.793		0.741		0.335		0.968		0.558
25 largest by revenue	77	103.7 ± 52.4; 98 (65-140.5)		50.1 ± 43.9; 37 (19-75.5)		7.3 ± 5.8; 6 (3-10.5)		46.3 ± 19.1; 45 (34-56)		153.9 ± 30.3; 147 (132-172)	
Other	51	101.9 ± 38.3; 93 (71-126)		48.7 ± 35.2; 40 (18-74)		6.3 ± 5.0; 6 (3-8)		46.9 ± 18.6; 44 (34-56)		152.0 ± 37.8; 141.5 (126.8-172.3)	

Values are presented as mean ± standard deviation; median (interquartile range). *P* values were calculated by Mann–Whitney and Kruskal–Wallis test.

ATMP, advanced therapy medicinal product; EPAR, European public assessment report.

Marketing authorisation type was significantly associated with the number of issues raised. Both CMAs (125.8 ± 43.1) and MAUECs (134.7 ± 22.7) elicited more rapporteur issues than standard procedures (95.2 ± 46.4). Expedited and supported pathways showed divergent effects, with accelerated assessment associated with fewer issues (72.5 ± 36.6), and EMA Priority Medicines with more (145.8 ± 72.6) compared to standard procedures (101.1 ± 40.9), particularly for quality-related issues.

### API and indication-related factors influencing marketing authorisation assessments

In addition to regulatory factors, the impact of pharmacochemical and clinically associated factors on marketing authorisation procedures was assessed (see Table 2). Analyses revealed a higher mean number of issues raised for biologic substances (114.7 ± 51.2) compared with small molecules (93.9 ± 41.1), driven by quality-related issues (65.1 ± 46.4 compared with 37.5 ± 30.6). Small molecules had a higher mean number of nonclinical issues (7.7 ± 5.4) compared with biologics (5.8 ± 4.6), while the number of clinical issues was comparable. Within biologics, ATMPs elicited significantly more issues (173.8 ± 69.2 compared with 103.4 ± 39.8), again primarily quality-driven (126.6 ± 60.0 compared with 53.4 ± 32.6).

**Table 2:** Summary of the number of issues raised (overall and per regulatory domain) and EPAR length, disaggregated by active substance type and indication-related attributes

	*N*	Number of issues raised	*P*	Thereof quality	*P*	Thereof nonclinical	*P*	Thereof clinical	*P*	Length of EPAR (pages)	*P*
Medicine type			0.011		<0.001		0.041		0.192		0.887
Small molecule	72	93.9 ± 41.1; 84 (61.8-125.8)		37.5 ± 30.6; 28 (15.3-49)		7.7 ± 5.4; 6 (4-10.8)		48.7 ± 19.9; 45 (36-58.5)		153.1 ± 30.0; 144 (132-169.8)	
Biologic	56	114.7 ± 51.2; 109 (82.5-144.8)		65.1 ± 46.4; 57 (26-90.5)		5.8 ± 4.6; 6 (3-8)		43.8 ± 17.1; 44 (32.5-52.3)		153.2 ± 37.2; 146.5 (128.3-176)	
ATMP (biologics)			<0.001		<0.001		0.204		0.272		0.697
Yes	9	173.8 ± 69.2; 143 (126-215.5)		126.6 ± 60.0; 95 (84.5-175)		8.7 ± 7.5; 6 (4-13)		38.6 ± 15.4; 39 (24-48)		150.6 ± 50.1; 146 (114-180.5)	
No	47	103.4 ± 39.8; 101 (76-141)		53.4 ± 32.6; 50 (23-77)		5.2 ± 3.8; 6 (2-8)		44.7 ± 17.4; 45 (34-53)		153.7 ± 34.8; 147 (129-176)	
FIC			0.624		0.472		0.958		0.606		0.421
Yes	41	103.7 ± 43.7; 70 (24-97)		51.8 ± 38.1; 40 (19-80.5)		6.8 ± 5.1; 6 (2-9.5)		45.1 ± 19.1; 44 (30.5-56)		150.7 ± 34.8; 140 (129-170.5)	
No	87	102.7 ± 48.9; 66 (33-98)		48.5 ± 41.8; 37 (18-35)		6.9 ± 5.2; 6 (3-10)		47.2 ± 18.7; 45 (35-56)		154.3 ± 32.5; 147 (130-171)	
Targeted therapy			0.336		0.102		0.872		0.236		0.141
Yes	54	98.6 ± 45.1; 86.5 (60.5-142.5)		42.7 ± 34.0; 32 (15-60.5)		6.8 ± 4.7; 6 (4-10)		49.1 ± 19.6; 45 (35.8-59.8)		157.7 ± 32.9; 148 (132.5-175)	
No	74	106.3 ± 48.6; 101 (74.5-129)		54.6 ± 44.2; 47 (19-76.3)		6.9 ± 5.5; 6 (3-10)		44.7 ± 18.1; 44 (34-53.3)		149.3 ± 33.0; 142.5 (126.5-166)	
Agnostic indication			0.042		0.290		0.217		0.013		0.006
Yes	3	152.7 ± 18.6; 144 (140-175)		60.0 ± 16.5; 52 (49-79)		10.7 ± 6.4; 8 (6-18)		82.0 ± 29.8; 77 (55-114)		210.0 ± 18.5; 203 (196-231)	
No	125	101.8 ± 47.0; 96 (66.5-130)		49.3 ± 41.0; 37 (18.5-74.5)		6.8 ± 5.1; 6 (3-10)		45.7 ± 17.8; 44 (34-56)		151.8 ± 32.3; 144 (130-169)	
No. of indications			0.650		0.496		0.985		0.022		0.004
1	113	102.7 ± 48.5; 93 (66.5-136)		51.1 ± 42.2; 37 (19-77)		6.9 ± 5.2; 6 (3-10)		44.8 ± 17.2; 44 (33-56)		149.9 ± 30.7; 143 (129.5-166)	
≥2	15	105.0 ± 36.2; 113 (71-125)		38.3 ± 23.0; 49 (16-56)		6.6 ± 5.0; 6 (3-10)		60.1 ± 24.5; 49 (44-77)		177.9 ± 41.3; 186 (144-206)	
Orphan designation			0.200		0.268		0.392		0.388		0.895
Yes	43	109.2 ± 44.1; 101 (79-143)		54.9 ± 43.1; 43 (23-82)		6.3 ± 4.8; 6 (3-8)		47.9 ± 18.7; 46 (37-60)		154.6 ± 34.6; 144 (129-170)	
No	85	99.9 ± 48.5; 93 (62.5-93)		46.9 ± 39.2; 35 (17-63)		7.2 ± 5.3; 6 (3-10)		45.9 ± 18.9; 44 (34-56)		152.4 ± 33.6; 146 (130-172.5)	

Values are presented as mean ± standard deviation; median (interquartile range). *P* values were calculated by Mann–Whitney and Kruskal–Wallis test.

ATMP, advanced therapy medicinal product; EPAR, European public assessment report; FIC, first in class.

Indication-related characteristics also influenced assessments. Tumour-agnostic indications were associated with a significantly higher mean number of overall issues raised (152.7 ± 18.6 compared with 101.8 ± 47.0), clinical issues raised (82.0 ± 29.8 compared with 45.7 ± 17.8), and EPAR length (210.0 ± 18.5 compared with 151.8 ± 32.3 pages). Applications covering multiple indications similarly elicited both more clinical issues raised and increased length of the EPAR. Procedures with more than one indication resulted in a higher number of clinical issues (60.1 ± 24.5) compared with those with a single indication (44.8 ± 17.2), as well as a longer EPAR length (177.9 ± 41.3 compared with 149.9 ± 30.7 pages). By contrast, orphan designation, FIC status, and targeted medicine classification did not significantly affect outcomes.

### Development of assessment measures over time

**Figure 2:**
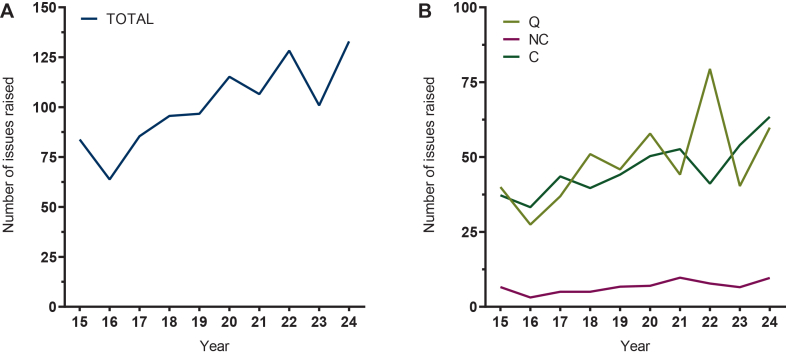
**Temporal developments in the number of distinct issues raised per procedure in the rapporteur’s initial assessment report from 2015 to 2024.** Overall (A) and by scientific domain (B): quality (Q), nonclinical (NC), and clinical (C). Values represent annual means.

**Figure 3:**
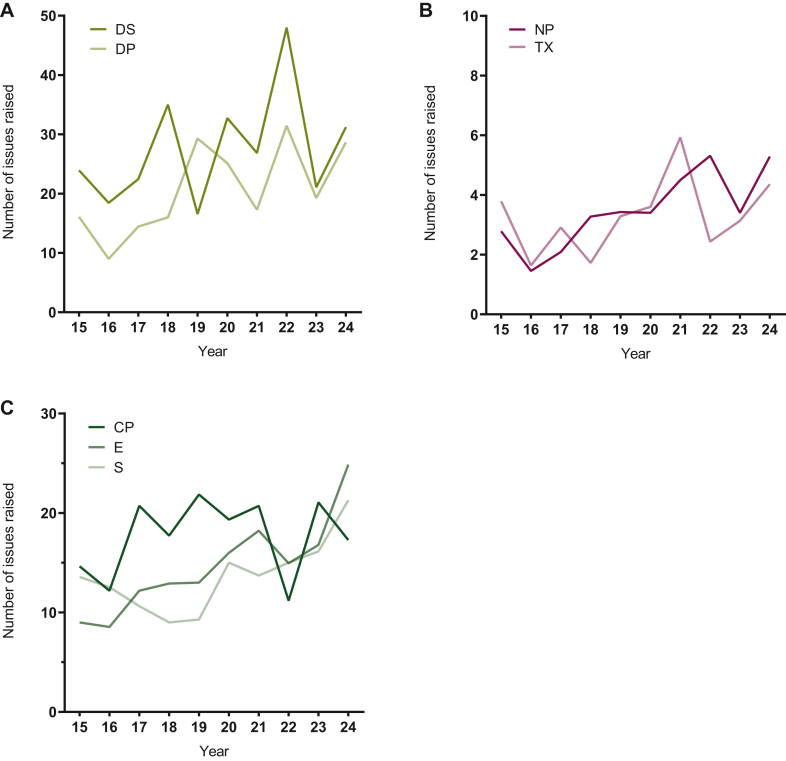
**Temporal developments in the number of distinct issues raised per procedure in the rapporteur’s initial assessment report, stratified by the three major scientific domains and their respectivesubdomains from 2015 to 2024.** (A) Quality, presented as issues related to drug substance (DS) and drug product (DP). (B) Nonclinical, presented as issues related to nonclinical PK/PD (NP) and toxicology (TX). (C) Clinical, presented as issues related to clinical PK/PD (CP), efficacy (E), and safety (S). Values represent annual means. PD, pharmacodynamics; PK, pharmacokinetics.

The number of distinct issues raised per procedure increased from 83.8 ± 41.6 in 2015 to 132.9 ± 35.5 in 2024, representing a 59.6% increase (Figure 2A). Across scientific domains, issues related to quality increased from 40.0 ± 28.7 to 59.9 ± 31.8 (+49.7%), nonclinical issues from 6.6 ± 5.9 to 9.6 ± 5.6 (+45.5%), and clinical issues from 37.2 ± 17.0 to 63.4 ± 16.6 (+70.4%; Figure 2B). Within quality-related issues, DP issues increased from 16.1 ± 9.7 to 28.7 ± 26.9 (+78.2%), and drug substance-related issues increased from 23.9 ± 23.1 to 31.2 ± 20.5 (+30.5%), demonstrating greater variability in the latter (Figure 3A). For nonclinical issues, an increase was observed in nonclinical PK/PD-related issues (2.8 ± 2.9 to 5.3 ± 3.5; +89.2%), while toxicology related issues remained relatively stable (3.8 ± 3.2 to 4.4 ± 3.1; +15.8%; Figure 3B). The increase in clinical-related issues was driven by efficacy, rising from 9.0 ± 7.7 in 2015 to 24.9 ± 8.9 in 2024 (+176.7%) and safety-related issues, increasing from 13.6 ± 7.2 to 21.3 ± 8.3 (+56.6%). Clinical PK/PD issues, despite considerable variability, showed no increase throughout the study period (14.6 ± 11.4 to 17.3 ± 8.1; +18.5%; Figure 3C). The length of the EPAR increased significantly over time, from 131.4 ± 20.0 pages in 2015 to 183.0 ± 29.7 pages in 2024 (39.2%; Supplementary Figure S2B, available at https://doi.org/10.1016/j.esmoop.2026.107732). After *z*-score normalisation the relative temporal changes in EPAR length mirrored those observed for the *z*-score normalised number of clinical issues raised (Supplementary Figure S3, available at https://doi.org/10.1016/j.esmoop.2026.107732). This correlation was less pronounced for the nonclinical and quality-related issues (data not shown). Procedure duration did not exhibit a significant trend throughout the observation period, with values of 342.1 ± 138.0 days in 2015 and 397.0 ± 130.3 days in 2024 (+16.1%), and limited variation around a mean of 357.0 ± 32.2 days (Supplementary Figure S1B, available at https://doi.org/10.1016/j.esmoop.2026.107732). All data are presented as numerical values, including mean ± SD and median (interquartile range), as detailed in Supplementary Table S2, available at https://doi.org/10.1016/j.esmoop.2026.107732.

## Discussion

The number of distinct issues offers a proxy for assessment complexity, rendering implicit dimensions of regulatory work more transparent and allowing their broader implications to be evaluated. Applied to oncology MAAs, this measure revealed substantial heterogeneity, reflecting differences in procedural pathways and product-specific characteristics. Most notably, a consistent increase in the number of distinct issues raised was observed across all domains and most subdomains over time. This rise was most pronounced in the clinical domain, particularly for efficacy-related issues, which more than doubled over the past decade. Although the exact factors are likely multifactorial, several contributors warrant discussion and closer examination.

The increase is plausibly associated with the intrinsic scientific complexity of innovative medicines. Sustained innovation, manifested through novel mechanisms of action, narrowly defined or heterogeneous patient populations, non-standard and adaptive study designs and emerging evidence paradigms such as real-world data,^9^, ^10^, ^11^ can substantially increase evaluative demands on assessors.

Further complexity arises as the evolving regulatory framework has led applicants to confront progressively more detailed requirements and an expanding body of guidance. The consequently growing comprehensiveness of submissions inevitably provides assessors with more material to evaluate and, where necessary, question. On the other hand, regulatory authorities themselves operate within an environment of continuously advancing assessment standards, reflected in the proliferation of guidelines and formalised assessment methodologies. Although these measures are meant to strengthen consistency and robustness of decision making, they also expand the scope of aspects to be reviewed, verified, and documented.

Other systemic factors likely amplify this trend. Globalisation of clinical trials and data generation introduce additional variability in methods and data quality, particularly in emerging areas such as real-world data or patient-reported outcomes.^12^^,^^13^ Organisational dynamics within both applicant companies and regulatory agencies, including assessor expertise, experience, and capacity, have been discussed to influence the volume and nature of regulatory questions, as some subjectivity remains in how concerns are documented and classified despite standardisation methods.^14^ Additional drivers have also been suggested, including trends towards corporate mergers or acquisitions, which may influence the organisation or presentation of submitted data, integration of external partnerships and resources, and evolving requirements from multiple stakeholders, such as health technology assessment bodies, which can contribute to procedural complexity or the need for supplementary information.^15^^,^^16^ Finally, evolving expectations for transparency and accountability have expanded documentation requirements, reinforcing the need for assessors to justify their evaluations and contributing to the growing number of questions.^17^ Although the observed increase in questions likely reflects the interplay of sustained scientific innovation, evolving regulatory frameworks, and broader systemic factors, the relative contribution of each element remains unclear. Nonetheless, considering these elements provides a framework to interpret the trends and their implications for regulatory practice and highlights the need for further research to inform the optimisation of regulatory processes and the strategic allocation of evaluative resources.

The increase in the number of issues raised underscores the growing complexity faced by regulators. However, the average number of initial MAA procedures and their review durations remained largely stable over time, indicating that efficiency was maintained despite escalating demands.^18^, ^19^, ^20^ Regulatory agencies have adapted through enhanced collaboration within the European medicines regulatory network. The introduction of multinational assessment teams has enabled broader involvement of national authorities, optimising resource use, while preserving high-quality scientific output.^21^ Initiatives such as IncreaseNet provide structured mentorship and on-the-job training, linking junior and senior colleagues to foster capacity development and trans-institutional knowledge exchange.^22^ For a better use of external expertise, including academic researchers and specialist clinicians, EMA and National Competent Authorities have completed a pilot enabling clinical oncology scientists to contribute to medicines regulation, without prejudice to the rigorous safeguards in place to ensure absence of conflicts of interest.^23^ What is more, efforts are underway to leverage artificial intelligence as an additional resource, aiming to manage the growing volume and complexity of data.^24^ Collectively, these measures demonstrate that regulators are actively navigating the rising assessment complexity, maintaining procedural efficiency while ensuring scientific scrutiny.

Although the overall rise in the number of issues signals increasing assessment complexity, the aggregate view inevitably conceals nuances. Regulatory pathways and product characteristics each impose distinct evaluative challenges. Disaggregating the data by these dimensions enables identification of systematic patterns behind the trend and highlights which product types or procedures may contribute to the overall rise.

The regulatory pathway was associated with differences in the number of issues raised. CMA and MAUEC applications elicited significantly more issues than standard Art 8(3) procedures, reflecting the lack of comprehensive clinical data at the time of authorisation. As a result, regulators required additional clarifications, particularly when CMA status is not proactively sought by the applicant but emerges as a consequence of the regulatory evaluation.^25^

Product type strongly influenced the number of issues. Biologics generally elicited more issues than small-molecule medicines, primarily driven by quality-related concerns, particularly DS issues, aligning with the complexity and requirements of biologically derived products. Among biologics, this is particularly evident for ATMPs, consistent with their individualised nature, sophisticated manufacturing processes and novel therapeutic modalities.^26^^,^^27^ Clinical issues, however, were broadly comparable across product types, indicating consistent efficacy and safety standards. Such regulatory consistency serves the paramount interest of patients, ensuring access to safe and efficacious treatments irrespective of the therapeutic complexity or novelty of the product. FIC medicines were not associated with a higher number of issues being raised, challenging the assumption that mechanistic novelty inherently entails greater regulatory uncertainty. Heterogeneity within pharmacological classes—arising from variations in therapeutic indications, molecular architecture, or clinical context—may limit comparability and transferability of assessments, suggesting that complexity arises from determinants other than the novel mechanism. Additionally, this finding may be explained by the fact that FIC products are often supported by extensive clinical programmes and shaped by early alignment with regulatory expectations through EMA scientific advice.^28^ Likewise, no relevant differences were observed for orphan medicines, indicating that, despite smaller patient populations, applicants generally provide sufficiently robust clinical data to support regulatory evaluation, comparable to non-orphan products receiving a positive CHMP opinion. This may similarly reflect effective presubmission alignment through EMA scientific advice and protocol assistance, a special form of advice available for developers of designated orphan medicines, as well as the impact of orphan incentives and integration of real-world data to mitigate residual uncertainty.^29^ Notably, oncology features highly standardised clinical endpoints and a relatively high volume of orphan procedures, further supporting comparability. These findings align with those of previous studies, reporting that neither FIC status nor orphan designation is associated with greater regulatory uncertainty in assessment reports.^30^ Targeted therapies exhibited a modest, nonsignificant increase in clinical issues, likely reflecting heterogeneity across indications. Genuinely novel biomarkers coupled with companion diagnostic-linked products pose far greater regulatory complexity than therapies directed at well-established molecular targets,^31^^,^^32^ potentially diluting sharper differences in aggregate analyses. Tumour-agnostic therapies exhibited a pronounced rise in clinical issues, indicative of the uncertainties linked to a paradigm shift in medicine that strains existing regulatory frameworks and established guidelines.^33^^,^^34^ Similarly, multi-indication products elicited more issues, consistent with the added complexity of evaluating expanded clinical programmes.

The length of the EPAR increased over time, in agreement with the rise in number of issues raised across domains of the application. EPAR length was selected as a complementary measure. In contrast to the initial assessment, the EPAR represents the final document of the MAA, capturing the definitive opinion and reasoning. This approach allows trends to be examined at both a very early and a very late procedural stage. Despite the temporal gap and potential procedural adjustments, including changes of indications, both measures generally reflect the same findings. However, as the EPAR primarily reflects the clinical module of the dossier, its length is particularly sensitive to complexity arising from pivotal phase III studies, whereas most quality-related and nonclinical data content remains confidential.^35^ Beyond issues recorded in the list of questions, the EPAR includes numerous formal and administrative sections, making it less responsive to submission-specific complexity than issue counts. Thus, EPAR length provides a pragmatic, albeit less precise, proxy measure of regulatory assessment complexity than issue counts. The strong alignment between the *z*-score of EPAR length and the number of clinical issues raised further demonstrates that the volume of assessor-raised issues is reflected in the public document, underscoring the transparency of the regulatory assessment process.

### Conclusion

The rising number of distinct issues raised during initial dossier assessment highlights a clear and sustained increase in regulatory assessment complexity in oncology marketing authorisations over the past decade. Despite this, European authorities have maintained stable capacity and timelines, underscoring their ability to manage the growing evidentiary complexity. The applied metrics capture both product-specific and systemic procedural influences, offering a quantitative lens on evolving regulatory challenges across quality, nonclinical, and clinical domains. These findings provide a granular evidence base to inform discussions on resource allocation and process optimisation. Systematic monitoring of assessment complexity can support evolving regulatory strategies, enabling regulators to maintain high scientific rigour and timely patient access to innovative therapies, despite the increasing complexity of a rapidly evolving science landscape.

## Supplementary data

Supplementary Tables S1-S2 and Figures F1-F3
