# Supplementary material for: Trends in EU regulatory assessment of oncology medicines
Source: ESMO Open. 2026 May 21;11(6):107732. doi: 10.1016/j.esmoop.2026.107732 (PMC13199759; doi:10.1016/j.esmoop.2026.107732)
Supplement: Supplementary Tables S1-S2 and Figures F1-F3 [file mmc1.pdf]

Supplementary Data:

Supp. Table 1: *List of the 25 largest pharmaceutical companies worldwide by total revenue, based on annual reports from 2024 (or, where unavailable, from 2023).*

Johnson & Johnson
China National Pharmaceutical Group Corporation (CNPGC) / Sinopharm
F. Hoffmann-La Roche AG
Merck & Co., Inc / Merck Sharp & Dohme
Pfizer Inc.
AbbVie Inc.
Bayer AG
AstraZeneca plc
Novartis AG
Bristol-Myers Squibb Company
Eli Lilly and Company
Sanofi S.A.
Abbott Laboratories
Novo Nordisk A/S
GSK plc
Shanghai Pharmaceuticals
Takeda Pharmaceutical Company Limited
Amgen Inc.
C.H. Boehringer Sohn AG & Co. KG
Gilead Sciences, Inc.
Merck Group
GE Healthcare Technologies, Inc.
Teva Pharmaceutical Industries Ltd.
Viatris Inc.
Regeneron Pharmaceuticals, Inc.

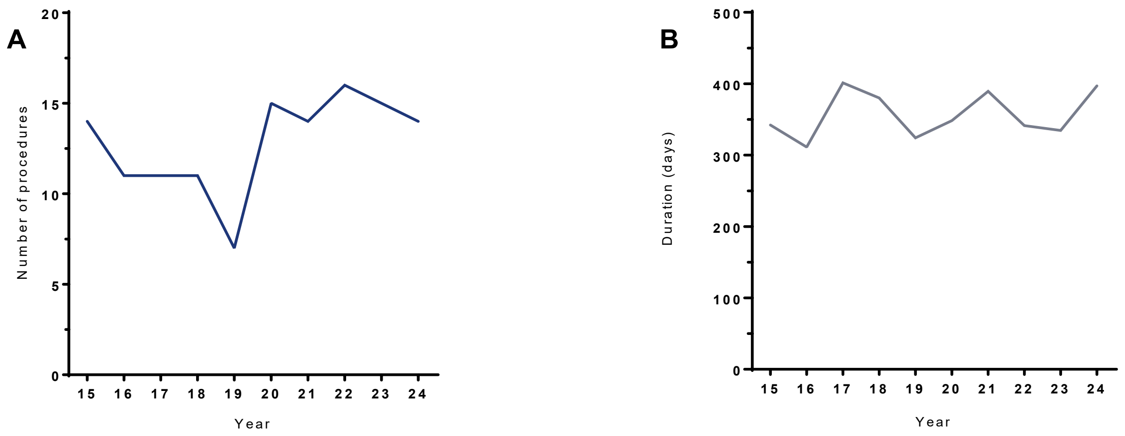

Supp. Figure 1: [A] *Number of positive opinions per year issued for centralised procedures concerning initial marketing authorisations of new chemical entities in oncologic medicines for human use, 2015–2024.* ***[B]*** *Temporal trends in procedure duration (days) per procedure from 2015 to 2024. Values represent annual means.*

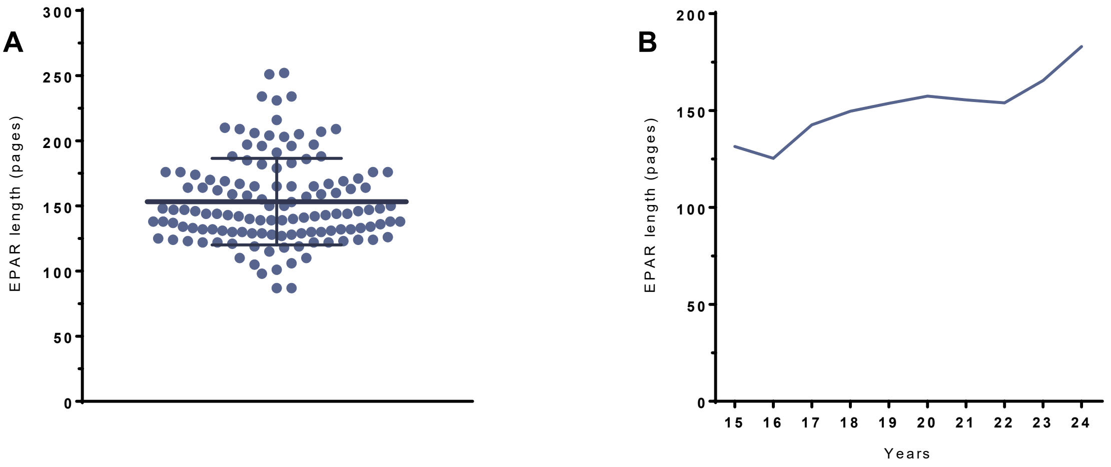

Supp. Figure 2: [A] *Total length of EPAR per procedure, expressed in number of pages. 152.2 ± 37.8; 146 (128.5-176). Values are presented as mean ± SD; median (IQR).* ***[B]*** *Temporal developments in the number of EPAR length (pages). Values represent annual means.*

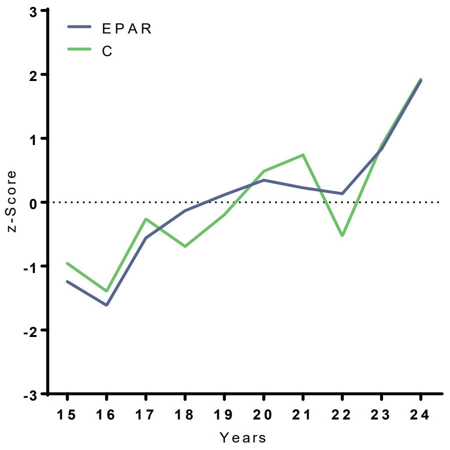

Supp. Figure 3: *Z-score–normalized temporal developments depicting relative year-to-year fluctuations in the number of EPAR pages (EPAR) and the number of clinical issues (C) raised in the rapporteur's initial assessment report from 2015 to 2024.*

Supp. Table 2: *Summary of the number of issues raised (overall and per scientific domain) and EPAR length, disaggregated by year of CHMP opinion. Values are presented as mean ± SD; median (IQR).*

Year	Total	Quality	Drug Substance	Drug product	Non-Clinical	Non-Clinical PKPD	Toxicology	Clinical	Clinical PKPD	Efficacy	Safety	EPAR
2015	83.8 ± 41.6; 75 (53-108.5)	40.0 ± 28.7; 33 (18-56.5)	23.9 ± 23.1; 15 (9-29.7)	16.1 ± 9.7; 14.5 (7.7-23.2)	6.6 ± 5.8; 5 (2.5-10.5)	2.8 ± 2.8; 2 (0.7-3.7)	3.8 ± 3.2; 3 (1.5-6.5)	37.2 ± 16.9; 33 (23.7-53.7)	14.6 ± 11.3; 12.5 (5.7-21)	9.0 ± 7.7; 6 (5-9.5)	13.6 ± 7.1; 12 (9.7-14.2)	131.4 ± 20.0; 131 (119.8-144)
2016	63.8 ± 41.8; 52 (32-76)	27.5 ± 37.9; 12 (10-29)	18.4 ± 33; 6 (4-15)	9.0 ± 7.5; 7 (5-14)	3.1 ± 2.7; 1 (1-6)	1.5 ± 1.6; 1 (0-3)	1.6 ± 1.8; 1 (0-2)	33.3 ± 24; 26 (18-36)	12.2 ± 6.9; 11 (8-16)	8.5 ± 7; 8 (3-15)	12.5 ± 12.1; 7 (5-13)	125.3 ± 19.4; 119 (110-140)
2017	85.5 ± 29.5; 78 (61-96)	36.9 ± 33.9; 24 (16-44)	22.4 ± 27.6; 11 (6-34)	14.5 ± 8; 12 (9-18)	5.0 ± 2.2; 4 (3-8)	2.1 ± 1.4; 2 (1-3)	2.9 ± 2.3; 2 (1-4)	43.5 ± 14.9; 48 (29-56)	20.7 ± 10.9; 21 (9-31)	12.2 ± 4.5; 11 (8-17)	10.6 ± 6; 10 (6-15)	142.7 ± 34.5; 131.5 (122.3-161.8)
2018	95.6 ± 50.3; 84 (64-117)	51.0 ± 48.3; 27 (16-77)	35.0 ± 39.5; 15 (8-50)	16.0 ± 11.3; 17 (5-25)	5.0 ± 4.4; 3 (1-8)	3.3 ± 2.7; 2 (1-6)	1.7 ± 2.2; 1 (0-2)	39.6 ± 7.8; 43 (35-44)	17.7 ± 10.4; 15 (10-29)	12.9 ± 8.9; 8 (5-25)	9.0 ± 3.8; 10 (6-11)	149.6 ± 25.9; 139 (132-171)
2019	96.7 ± 35.1; 82 (78-140)	45.9 ± 35.3; 37 (21-79)	16.8 ± 9.5; 16 (13-22)	29.3 ± 33.7; 15 (5-78)	6.7 ± 4.2; 6 (4-9)	3.4 ± 2; 3 (2-5)	3.3 ± 2.8; 4 (1-5)	44.1 ± 13.6; 39 (32-55)	21.9 ± 6.3; 23 (16-27)	13.0 ± 6.7; 12 (7-22)	9.3 ± 3.6; 7 (6-13)	153.7 ± 22.3; 148 (140-163)
2020	115.2 ± 36.1; 120 (85-142)	57.9 ± 33.5; 52 (28-81)	32.7 ± 23.9; 22 (10-61)	25.1 ± 14.9; 23 (15-32)	7.0 ± 3.2; 6 (5-10)	3.4 ± 2.4; 4 (1-5)	3.6 ± 2.3; 3 (2-5)	50.3 ± 24.5; 45 (39-53)	19.3 ± 9.4; 19 (14-28)	16.0 ± 17.8; 11 (7-16)	15.0 ± 8.4; 16 (8-19)	157.5 ± 33.7; 144 (131-174)
2021	106.5 ± 38.4; 108.5 (73.5-136)	44.1 ± 29.5; 45.5 (16.5-74.7)	26.9 ± 21.9; 23 (7-43.2)	17.3 ± 11.8; 16 (7.7-25)	9.7 ± 6.6; 8 (5.7-14.5)	4.5 ± 3.5; 3.5 (1.7-6.7)	5.9 ± 5.5; 3.5 (1.5-11.7)	52.6 ± 14.8; 51.5 (38.5-64.5)	20.7 ± 8.1; 22.5 (14-25.2)	18.2 ± 8.4; 19.5 (13-23.5)	13.7 ± 8.8; 11.5 (9-14.2)	155.5 ± 19.9; 153 (138.8-164.3)
2022	128.3 ± 71.1; 116.5 (80.2-146.8)	79.4 ± 63.5; 65.5 (29.7-94.5)	48.0 ± 47.8; 30 (17-71.2)	31.4 ± 20.4; 27 (15-44.5)	7.8 ± 5.3; 6 (5.2-10.7)	5.3 ± 4.8; 4 (2-7.7)	2.4 ± 1.3; 2 (1.2-3.7)	41.1 ± 13.8; 42.5 (28-49)	11.2 ± 6.9; 10.5 (6-16)	14.9 ± 8.2; 13 (10.2-19)	15.0 ± 5.6; 15 (11-20.2)	154 ± 38.0; 152 (133.3-176)
2023	100.9 ± 39.6; 98 (53-148)	40.3 ± 31.9; 33 (14-56)	21.1 ± 21.4; 17 (5-38)	19.3 ± 13; 18 (7-26)	6.5 ± 5.6; 5 (1-10)	3.4 ± 2.9; 3 (0-6)	3.1 ± 3.6; 1 (0-7)	54.0 ± 16.5; 48 (43-66)	21.1 ± 11.4; 20 (15-27)	16.8 ± 7.3; 17 (13-21)	16.1 ± 11.1; 14 (6-23)	165.5 ± 41.2; 155 (130-197)
2024	132.9 ± 32.5; 135.5 (111-154.3)	59.9 ± 31.1; 52.5 (32.5-83.7)	31.2 ± 20.4; 29.5 (13.5-44)	28.6 ± 26.9; 21 (13-33.7)	9.6 ± 5.5; 9 (6-14)	5.3 ± 3.4; 5 (2.7-8)	4.4 ± 3.1; 4.5 (2-6)	63.4 ± 16.6; 59.5 (51.2-74.7)	17.3 ± 8; 17 (9.5-23.5)	24.9 ± 8.8; 21 (18.7-33.5)	21.2 ± 8.3; 22.5 (15-27)	183 ± 29.7; 184 (162.3-203.3)
